# Impact of a major cardiovascular surgical procedure on patients' interests for advance care planning

**DOI:** 10.1186/2197-425X-3-S1-A650

**Published:** 2015-10-01

**Authors:** F Gigon, C Combescure, P Merlani, B Ricou

**Affiliations:** University Hospitals of Geneva, Geneva, Switzerland; University of Geneva, Geneva, Switzerland; Ospedale Regionale di Lugano, Lugano, Switzerland

## Introduction

Advance directives (AD) and/or a health care surrogate decision maker (HCS) are potentially helpful for caregivers to respect the patients' autonomy whenever their competence is affected. Patients planned for major cardiovascular surgical procedure requiring intensive care may consider AD/HCS as important or necessary because of the coming exposure to a potential life-threatening situation.

## Objectives

To investigate whether a major cardiovascular surgical procedure requiring intensive care impacts on patients', interests for AD/HCS.

## Methods

Patients planned for major cardiovascular surgery were randomized either to a group A (**gA**) met the day **before** and **after** ICU discharge, or to a group B (**gB**) met only **after** ICU discharge. At each meeting, they were interviewed according to the same questionnaire.

## Results

361(89%) patients (of 405 eligible) were interviewed. Male: 256(71%); age(mean ± SD):68 ± 15 years. 95(27%) had a last will, 77(21%) a life insurance, 119(33%) a funeral plan and 43(12%)an organ donor card. 181(50%) patients were randomized in the gA, 180(50%) in the gB.

After surgery, 164(91%) of the gA patients remembered the interview - before, 90(50%) what AD are and 61(34%) could give a correct definition of AD.

## Conclusions

Few patients, even when scheduled for major surgical procedure, knew what AD or HCS are and even fewer had AD/HCS. Their incidence was much lower than other plans for the future (last will, life insurance, etc.). Undergoing major surgery requiring intensive care modified significantly the attitudes of patients towards AD/HCS, decreasing their interest. Further analyses regarding these patients' reasons for or against AD/HCS will provide more information to understand the rarity of advance care planning.

## Grant Acknowledgment

This study is sustained by the FNRS (CR31I3_127135/1)

**Figure 1:**
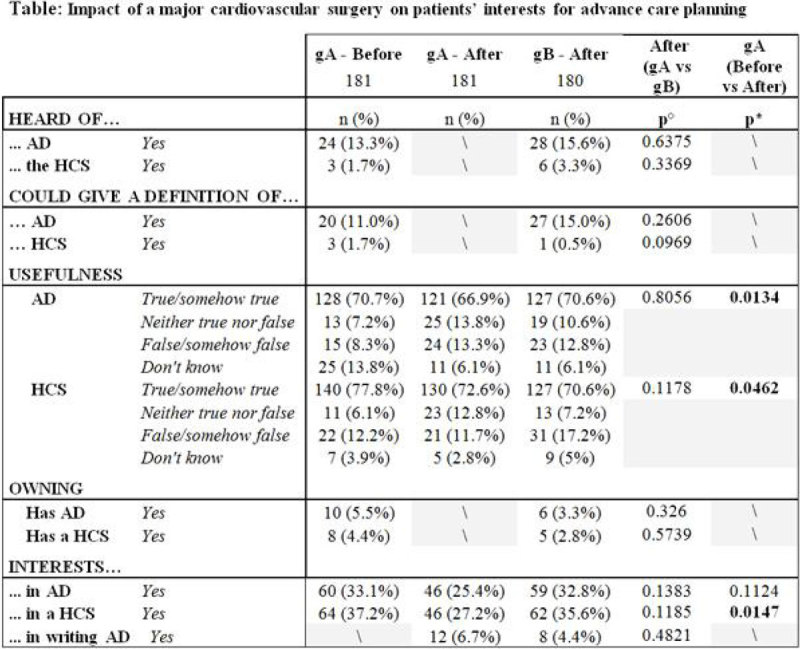
**Figure legends: p°: Chi** ^**2**^ , p*: Mc Nemar, \: NA

